# Fenugreek (Trigonella Foenum-Graecum) Seed Flour and Diosgenin Preserve Endothelium-Dependent Arterial Relaxation in a Rat Model of Early-Stage Metabolic Syndrome

**DOI:** 10.3390/ijms19030798

**Published:** 2018-03-10

**Authors:** Katalin Szabó, Rudolf Gesztelyi, Nóra Lampé, Rita Kiss, Judit Remenyik, Georgina Pesti-Asbóth, Dániel Priksz, Zoltán Szilvássy, Béla Juhász

**Affiliations:** 1 Department of Pharmacology and Pharmacotherapy, Faculty of Medicine, University of Debrecen, Nagyerdei krt. 98, H-4032 Debrecen, Hungary; szabo.katalin@pharm.unideb.hu (K.S.); gesztelyi.rudolf@pharm.unideb.hu (R.G.); lampenori@gmail.com (N.L.); kiss.rita@med.unideb.hu (R.K.); priksz.daniel@pharm.unideb.hu (D.P.); szilvassy.zoltan@med.unideb.hu (Z.S.); 2 Institute of Food Technology, Faculty of Agricultural and Food Sciences and Environmental Management, University of Debrecen, Boszormenyi ut 138, H-4032 Debrecen, Hungary; remenyik@agr.unideb.hu (J.R.); georgina.asboth@gmail.com (G.P.-A.)

**Keywords:** endothelial dysfunction, metabolic syndrome, obesity, type 2 diabetes mellitus, fenugreek, Trigonella foenum-graecum, diosgenin, endothelium-dependent vasorelaxation, Wistar rat

## Abstract

Fenugreek is a common herb possessing several bioactive components including diosgenin. Here, dietary fenugreek seed flour and diosgenin were evaluated on a model of endothelium-dependent vasorelaxation by abdominal aortas isolated from rats receiving high-fat, high-sugar diet (HFHSD). 60 male Wistar rats were randomized into six groups: (i) negative control getting conventional rat feed regimen; (ii) positive control receiving HFHSD; (iii) a test group fed 2 g/kg bw/day fenugreek seed flour (containing 10 mg/kg bw/day diosgenin) + HFHSD; (iv) three test groups fed 1, 10 and 50 mg/kg bw/day diosgenin + HFHSD. Alimentary treatments were carried out for six weeks. The abdominal aortas were isolated, and 2 mm wide rings were sectioned off and mounted at a resting tension of 10 mN in organ baths containing Krebs solution (36 °C) exposed to 95% O_2_ and 5% CO_2_. After 60-min incubation, a norepinephrine concentration-response (E/c) curve was generated to determine their half-maximal effective concentration (EC_50_) value. After 60-min wash-out, a pre-contraction with norepinephrine EC_50_ was made, followed by an acetylcholine E/c curve. Plasma glutathione levels, glutathione-handling enzyme activities and blood antioxidant capacities were also determined. HFHSD significantly decreased the dilatory response to acetylcholine and increased plasma glutathione levels and these effects were significantly reversed by fenugreek seed flour, 10 and 50 mg/kg bw/day diosgenin. Both fenugreek and diosgenin treatments prevent HFHSD-induced endothelial dysfunction and redox changes. As fenugreek treatment was more effective at lower acetylcholine concentrations than diosgenin treatments, components of fenugreek other than diosgenin may contribute to the beneficial effects of dietary fenugreek seed flour.

## 1. Introduction

Metabolic syndrome (MetS) is a group of metabolic disorders characterized by the presence of at least three of five pathological conditions, described as follows: central obesity, hypertension, hyperglycemia, hypertriglyceridemia and decreased level of high-density lipoprotein (HDL) [1,2]. MetS is one of the most prevalent and rapidly increasing challenges to public health worldwide, with onset and pathogenesis associated with frequent sedentary lifestyle choices and high calorie intake in affluent societies. MetS patients have a five-fold higher risk of developing type 2 diabetes mellitus (T2DM) and two-fold risk for cardiovascular diseases (CVD) than the general human population [3,4]. A major commonality of these pathologies is that they all engender impairment of endothelial function. Endothelial dysfunction (ED) forms the basis of atherosclerosis, which is the most important risk factor for CVD [5,6].

Although the vascular endothelium consists only of a thin monolayer of cells covering the inner surface of blood vessel wall, this tissue plays a crucial role in maintenance of a normal circulation. Beyond its barrier function, endothelium acts as a paracrine organ producing a number of transmitter molecules that contribute to the regulation of a wide range of processes including hemostasis, inflammation, vascular proliferation and vascular tone [7,8]. Endothelial cells synthesize both vasodilators (such as endothelium-derived relaxing factor (nitric oxide: NO), endothelium-derived hyperpolarizing factor, prostacyclin (PGI2)) and vasoconstrictor compounds (e.g., endothelins and leukotrienes) [9,10]. Certain metabolic, hormonal and homeostatic conditions, including hyperglycemia, hyperinsulinemia, hypertension, obesity, decreased level of high-density lipoprotein cholesterol (HDL), increased levels of insulin-like growth factor 1, fatty acids, triglycerides, low-density lipoprotein cholesterol (LDL), angiotensin II and plasminogen activator inhibitor-1, impair the barrier and regulatory functions of endothelial cells and thereby lead to ED. A major consequence of ED is that the balance between vasoconstriction and vasorelaxation is disrupted resulting in detrimental vasoconstriction that progresses into hypertension [11]. A further consequence of ED is a concomitant disharmony between coagulation and fibrinolysis, which can lead to enhanced thrombus formation. These deleterious processes, when added to other effects of hypertension, create conditions that favor atherosclerosis and elevated risk for cardiovascular events, especially stroke and acute myocardial infarction [12]. The most important underlying molecular mechanisms leading to ED are decreased production and/or increased degradation of NO, diminished responsiveness to NO, depletion of NADPH, increased NADH/NAD^+^ ratio, elevated levels of protein kinase C and advanced glycation end products; in general: increased oxidative stress and decreased antioxidant capacities [13].

The complexity of overlapping signaling pathways leading to ED has made identification of the primary triggering events problematic. A schematic representation of signaling pathways contributing to ED, made by Hsueh et al., provided enhanced clarity by delineating these processes in relation to progression of insulin resistance. This graphic construct suggests that hyperinsulinemia may act as a “zero point” event, which alters endothelial function in ways that progress into MetS. MetS, especially with obesity, increases systemic inflammation, oxidation of NO and LDL cholesterol, thereby increasing the risk of thrombosis, a condition that also adversely affects endothelial function. MetS is associated with impaired glucose tolerance and T2DM onset, two conditions that increase the risk of atherosclerosis [14].

In the present report, fenugreek seed flour was investigated as a possible countermeasure to the above-mentioned pathological changes, with special regard to the restoration of endothelial function. The rationale for selecting this plant is based on its “generally recognized as safe” (GRAS) status as a component of human diet, along with its known antidiabetic properties and wide availability. Fenugreek (*Trigonella foenum-graecum*) is one of the most commonly used medicinal herbs in the prevention and treatment of T2DM in many East Asian cultures. A component of this plant, diosgenin, along with 4-isohydroxyleucin and galactomannan, are the components thought to be the most responsible for the beneficial effects of fenugreek on MetS and T2DM [15]. Diosgenin is a phytosteroid sapogenin that can be chemically converted into several medically used steroid products, notably progesterone, pregnenolone and cortisone. Although metabolic effects of fenugreek have been widely studied, little definitive data has been published regarding its effects on the vasculature. It has, nevertheless, been noted that aqueous extracts of fenugreek leaf [16] and alcoholic extracts of fenugreek seed [17] were found to have beneficial effects in a streptozotocin-induced diabetic rat model. In contrast to fenugreek, several investigations dealt with diosgenin, primarily focusing on its ability to improve the condition of vasculature and/or endothelium in diabetic rats [18,19,20,21,22,23,24]. However, the underlying cellular and molecular mechanisms concerning diosgenin’s effects have remained to be defined at the time of this writing.

For the present investigation, a rat model of early-stage MetS was used. Wistar rat is a widely used experimental model of the disturbed carbohydrate and/or lipid metabolism [25,26].

## 2. Results

### 2.1. Characteristics of the Animal Model

**Figure 1:**
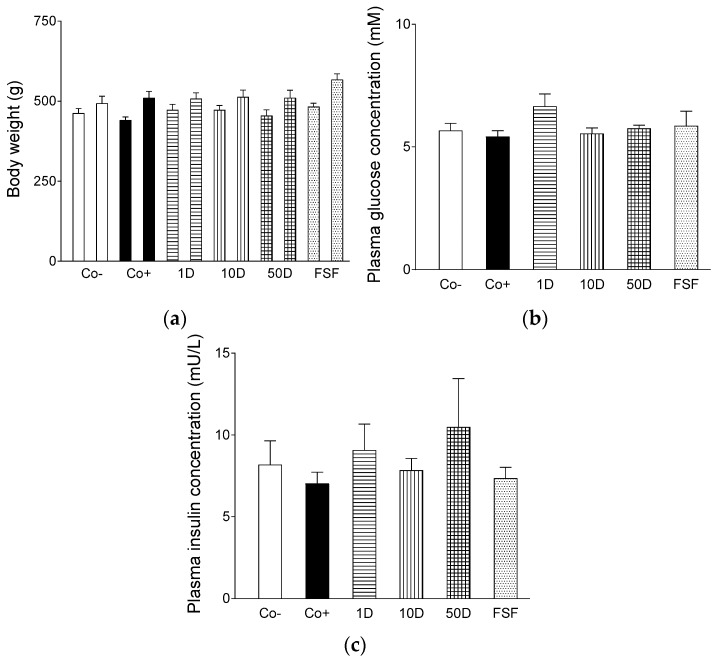
The effect of high-fat, high-sugar diet (HFHSD) (positive control: Co+), 2 g/kg bw/day fenugreek seed flour in addition to HFHSD (FSF), 1, 10 and 50 mg/kg bw/day diosgenin in addition to HFHSD (1D, 10D and 50D, respectively) on the body weight (**a**), fasting plasma glucose level (**b**) and fasting plasma insulin level (**c**) of Wistar rats. The negative control group (Co−) consisted of rats receiving conventional rat food and tap water. In the panel (**a**), the left one of columns with the same color and pattern indicates the initial body weight, while the right one shows the body weight after the 6-week dietary treatment. The columns represent the average values of the given groups (+SEM).

During the 6-week dietary treatment, rats showed a moderate (and statistically non-significant) body weight gain in every experimental group. The average weight gain was the biggest in the fenugreek group (treated with fenugreek seed flour: 85 g) followed by the positive control group (received HFHSD only: 69.9 g), 50 mg diosgenin group (got 50 mg/kg bw/day diosgenin: 55.9 g), 10 mg diosgenin group (received 10 mg/kg bw/day diosgenin: 40.6 g) and 1 mg diosgenin group (got 1 mg/kg bw/day diosgenin: 35.4 g), while the body weight gain was the smallest in the negative control group (kept conventionally: 30.9 g) (Figure 1a).

Neither fasting plasma glucose concentrations nor fasting plasma insulin levels, measured after the six-week dietary treatment, differed significantly among the groups (Figure 1b,c).

These results indicate that, in this model, no obesity or insulin resistance, two important pathological conditions characteristic of MetS, developed. Consistently, no significant effect of alimentary fenugreek or diosgenin treatment on the body weight and glucose homeostasis could be observed.

### 2.2. Response to Norepinephrine

**Figure 2:**
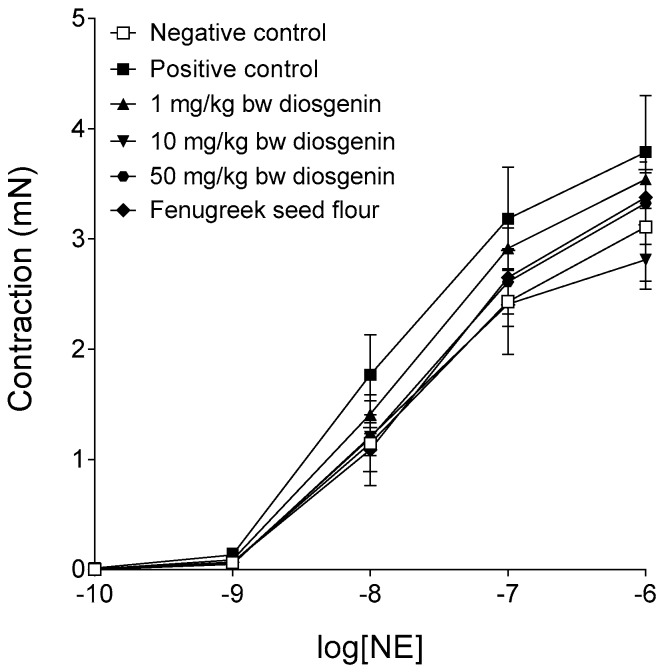
The effect of norepinephrine (NE) on the abdominal aorta isolated from rats treated with dietary fenugreek seed flour or different doses of diosgenin in addition to high-fat, high-sugar diet (HFHSD), and from rats forming the positive (receiving HFHSD only) and negative (getting conventional rat food and tap water) controls. The axis *x* shows the common logarithm of molar concentration of NE, while the axis *y* indicates the contractile force of the aortic rings (over the resting level). The length of the rings was the same throughout the study. The symbols represent the effect of NE averaged within the groups (±SEM).

The maximal contractile forces that evolved in the presence of the different NE concentrations did not differ significantly among the six groups (Figure 2). The statistically non-significant increase in the NE-susceptibility of the positive control group (subjected to HFHSD only) might indicate a moderate decrease in the basal production and/or availability of NO in comparison with that/those of the negative control group (encompassing rats kept conventionally). If that were the case, the applied alimentary fenugreek and diosgenin treatments might slightly improve vascular NO homeostasis (Figure 2).

### 2.3. Response to Acetylcholine

**Figure 3:**
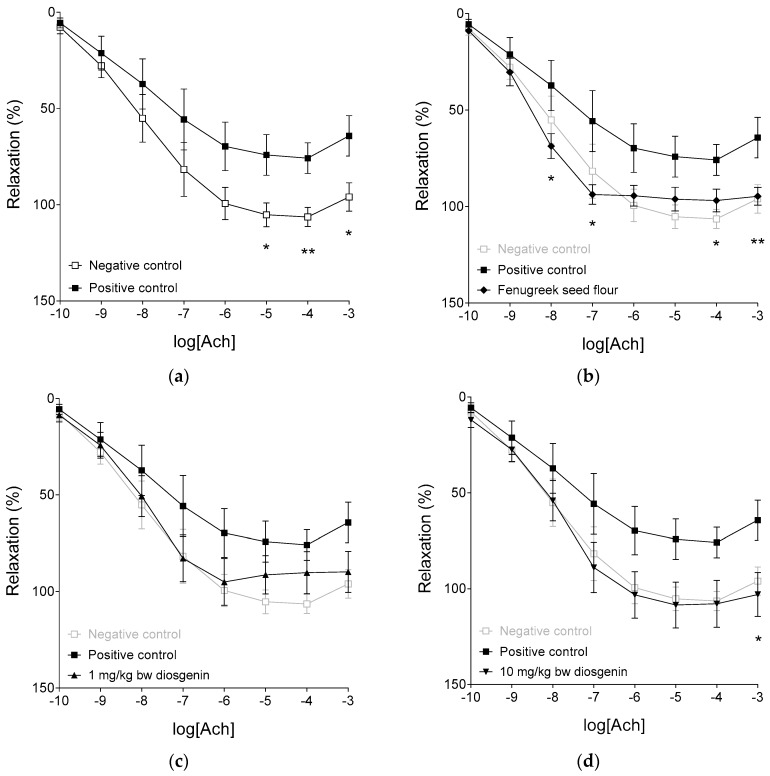

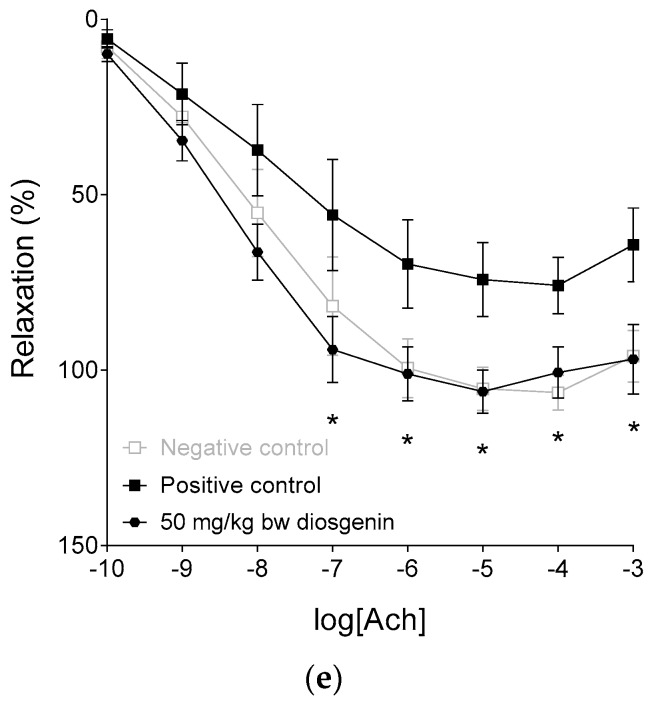
The effect of acetylcholine (Ach) on the abdominal aorta isolated from rats treated with dietary fenugreek seed flour (panel (**b**) or different doses of diosgenin in addition to high-fat, high-sugar diet (HFHSD) (panel (**c**–**e**)), and from rats forming the positive (receiving HFHSD only) and negative (receiving conventional rat food and tap water) controls (panel (**a**)). All aortic rings underwent a pre-contraction evoked by norepinephrine (NE) before the administration of Ach. The axis *x* shows the common logarithm of molar concentration of Ach, while the axis *y* indicates the effect as a percentage decrease of the initial contractile force of aortic rings. The symbols represent the effect of Ach averaged within the groups (±SEM). Asterisks denote the significance level of differences between groups indicated with black colored symbols and lines (*: *p* < 0.05; **: *p* < 0.01).

In the negative control group (consisting of rats kept conventionally), 10–100 µmol/L acetylcholine (Ach) could totally abolish the pre-contraction of aortic rings elicited by NE (used in EC_50_ concentration). In contrast, responses to Ach in the positive control group (receiving HFHSD only) showed a significant decrease (at 10, 100 and 1000 µmol/L Ach concentrations) when compared with those in the negative control group (Figure 3a). Thus, despite the absence of obesity and insulin resistance reflected by the results presented above (Figure 1), our HFHSD rat model displayed impaired endothelial function characteristic of disturbed carbohydrate and/or lipid metabolism.

The 2 g/kg bw/day fenugreek seed flour treatment significantly improved the response to Ach (even at relatively low Ach concentrations), almost totally preventing the deteriorative effect of HFHSD (Figure 3b). Treatment with diosgenin, a fenugreek component thought to be important regarding the beneficial effects of fenugreek preparations, can fairly mimic the action of fenugreek seed flour treatment in the present model. Although 1 mg/kg bw/day diosgenin failed to reach a statistically significant beneficial effect (due to the high scatter in the results reflecting very different individual responses to Ach in the 1 mg diosgenin group) (Figure 3c), 10 and 50 mg/kg bw/day diosgenin significantly augmented the Ach-induced relaxation (especially at medium and high Ach levels) in comparison with the positive control group (Figure 3d,e). Nevertheless, responses to Ach in the fenugreek and diosgenin groups did not differ from one another in a statistically significant manner.

It should be noted that the effect of 2 g/kg bw/day fenugreek seed flour treatment (equivalent to 10 mg/kg bw/day diosgenin treatment regarding its diosgenin content) showed two interesting differences in comparison with the action of diosgenin treatments. First, the shape of Ach concentration-response (E/c) curve was the least regular in the fenugreek group, probably owing to the complex effects afforded by the numerous components of fenugreek seed flour. Second, only fenugreek treatment could significantly enhance the relaxation at 10 nmol/L Ach concentration (a relatively low value), although the maximal relaxation evoked by Ach in the fenugreek group was (non-significantly) smaller than that in the negative control and diosgenin groups. It is also interesting that average effects of the different diosgenin treatments on the response to Ach were quite similar to one another, the increase of diosgenin dose predominantly reduced the scatter of the results, i.e., it homogenized the response to Ach. For further evaluation of the Ach E/c curves, see Appendix A.

### 2.4. Results of Assays for Blood Redox Status

**Figure 4:**
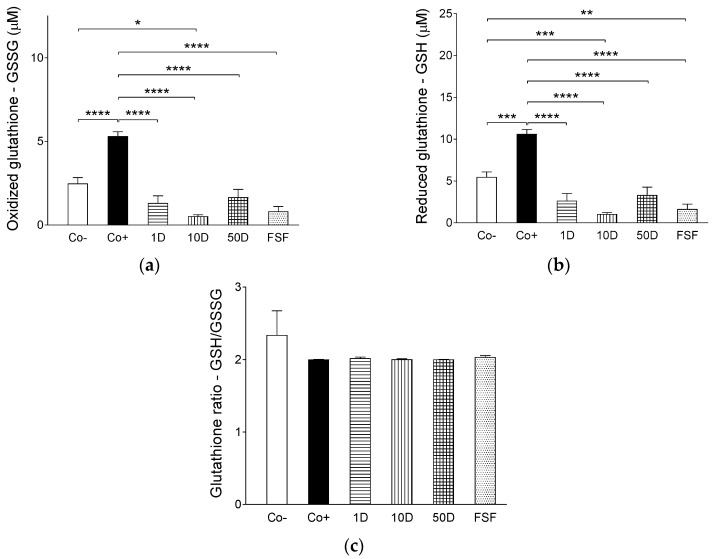
The effect of high-fat, high-sugar diet (HFHSD) (positive control: Co+), 2 g/kg bw/day fenugreek seed flour in addition to HFHSD (FSF), 1, 10 and 50 mg/kg bw/day diosgenin in addition to HFHSD (1D, 10D and 50D, respectively) on plasma levels of the oxidized (**a**) and reduced (**b**) forms of glutathione, furthermore on the ratio of reduced to oxidized glutathione (**c**) in Wistar rats. The negative control group (Co−) consisted of rats receiving conventional rat food and tap water. The columns represent the average values of the given groups (+SEM). Asterisks indicate the significance level of differences between groups (*: *p* < 0.05; **: *p* < 0.01; ***: *p* < 0.001; ****: *p* < 0.0001).

Results of the present investigation concerning the changes in plasma glutathione levels, glutathione-handling enzyme activities and antioxidant capacity in response to the different dietary treatments allow us to make some assumptions. The HFHSD tended to shift the oxidoreductive balance to the oxidative direction in the blood, indicated by an increase in oxidized glutathione level of positive control group (receiving only HFHSD) as compared to the negative control group (getting normal diet) (Figure 4a). Probably as a result of compensatory processes, the oxidative stress caused by HFHSD also increased the level of the reduced form of glutathione in the plasma of positive controls (Figure 4b) that led to a practically unchanged reduced to oxidized glutathione ratio (Figure 4c). It is clearly seen, furthermore, that the treatment with either diosgenin or fenugreek dramatically decreased the concentration of glutathione, equally affecting both of its forms (Figure 4). This phenomenon could also be considered as a result of counteracting processes against oxidative stress caused by HFHSD, if assuming that diosgenin and some compounds of fenugreek (including diosgenin itself) acted as effective antioxidants rendering sufficient oxidoreductive balance even at low glutathione plasma levels.

**Figure 5:**
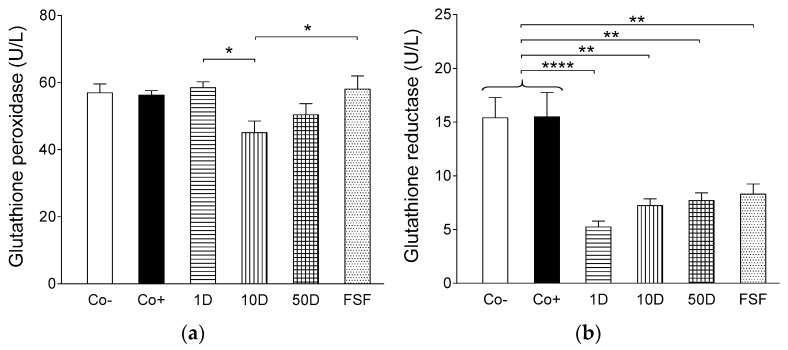
The effect of high-fat, high-sugar diet (HFHSD) (positive control: Co+), 2 g/kg bw/day fenugreek seed flour in addition to HFHSD (FSF), 1, 10 and 50 mg/kg bw/day diosgenin in addition to HFHSD (1D, 10D and 50D, respectively) on plasma activities of two major glutathione-handling enzymes, glutathione peroxidase (**a**) and glutathione reductase (**b**), in Wistar rats. The negative control group (Co−) consisted of rats receiving conventional diet. The columns represent the average values of the given groups (+SEM). Asterisks indicate the significance level of differences between groups (*: *p* <0.05; **: *p* < 0.01; ****: *p* < 0.0001).

**Figure 6:**
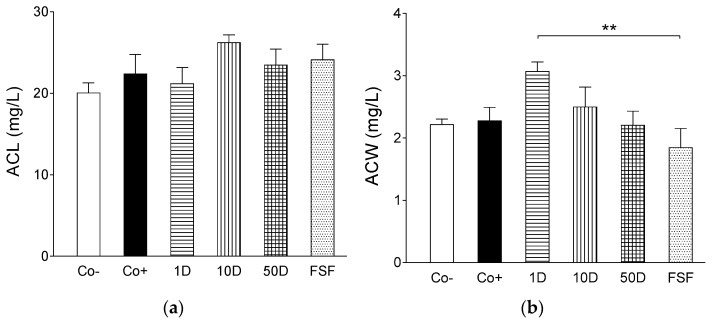
The effect of high-fat, high-sugar diet (HFHSD) (positive control: Co+), 2 g/kg bw/day fenugreek seed flour in addition to HFHSD (FSF), 1, 10 and 50 mg/kg bw/day diosgenin in addition to HFHSD (1D, 10D and 50D, respectively) on the antioxidant capacity of lipid- (ACL; (**a**)) and water-soluble (ACW; (**b**)) substances of blood in Wistar rats. The negative control group (Co−) consisted of rats receiving conventional rat food and tap water. The columns show the average values of the given groups (+SEM). Asterisks denote the significance level of differences between groups (**: *p* < 0.01).

Although activities of glutathione peroxidase, an enzyme which reduces the peroxide (O–O) moiety to an O–H moiety via oxidizing the reduced form of glutathione, did not show considerable differences among the groups (Figure 5a), activities of glutathione reductase, which regenerates the oxidized glutathione to a reduced one, proved to be significantly lower in the diosgenin- and fenugreek-treated groups as compared to both the negative and positive controls (Figure 5b). This latter finding corroborates the assumption described above, i.e., diosgenin and perhaps some other fenugreek compounds are effective antioxidants that decrease the need for glutathione. The counterbalancing function of glutathione (and possibly other endogenous oxidoreductive) system(s), which seem(s) to maintain a dynamic functional equilibrium with exogenous factors (e.g., dietary diosgenin), may be reflected in the finding that the antioxidant capacities of lipid- (Figure 6a) as well as water-soluble (Figure 6b) substances of the blood did not differ in a considerable way among the experimental groups receiving different dietary treatments.

In summary, diosgenin (and maybe some other components of fenugreek seed) appear(s) to substitute the endogenous antioxidant factors (including glutathione) to maintain the vascular redox balance. So, changes in endogenous antioxidant mechanisms (Figure 4, Figure 5 and Figure 6) can be considered to be rather compensatory responses to the presence of exogenous antioxidants than causes of the improvement of Ach responsiveness seen after addition of diosgenin or fenugreek (Figure 3). It means that diosgenin (and possibly some other fenugreek components) was/were more potent in preserving the endothelium-dependent vasorelaxation during HFHSD than the endogenous antioxidant factors.

## 3. Discussion

A primary outcome of the present study is that 2 g/kg bw/day dietary fenugreek seed flour, and 10 and 50 mg/kg bw/day dietary diosgenin (all applied in addition to HFHSD) practically completely prevented ED produced by HFHSD alone. Furthermore, fenugreek seed flour appears to augment endothelium-dependent vasorelaxation at lower Ach levels than diosgenin, suggesting that components of fenugreek other than diosgenin might contribute to the beneficial effect of fenugreek seed flour. It has been also found that HFHSD alone markedly increased the plasma levels of both oxidized and reduced glutathione, while this treatment did not significantly affect the activity of either glutathione peroxidase or glutathione reductase. In contrast, both fenugreek and diosgenin caused a dramatic decrease in plasma levels of both oxidized and reduced glutathione, with a simultaneous remarkable attenuation of glutathione reductase (but not glutathione peroxidase) activity. Interestingly, the blood antioxidant capacity has been found relatively insensitive to the different feed regimens used in the present study, thus, underscoring the efficiency and flexibility (and relative resistance to dietary factors) of processes that maintain redox balance in the blood. These results suggest that antioxidant agents within fenugreek seed (especially diosgenin) can modify the regulatory pathways underlying the redox balance of the blood (in a complex manner) that may contribute to the beneficial effect of these compounds on endothelium-dependent arterial dilatory response under disrupted metabolic conditions. In addition, our results have confirmed the previous observation that ED is an early sign of disturbed carbohydrate and lipid metabolism. Moreover, our findings indicate that a decrease in the endothelium-dependent arterial relaxation caused by HFHSD can precede the manifest hyperglycemia and hyperinsulinemia.

NO, a main vasodilatory substance, is generated through conversion of l-arginine to l-citrulline by three types of NO-synthases (NOSs): neuronal (nNOS or NOS1), inducible (iNOS or NOS2) and endothelial (eNOS or NOS3) ones. Endothelium expresses eNOS, while vascular smooth muscle was reported to contain all three NOS isoforms [27]. In the vascular smooth muscle cells, NO activates the guanylate cyclase causing an increase in cGMP level and consequent activation of protein kinase G that induces vasorelaxation [28]. Beyond this classic effect, activating the protein kinase B (Act) pathway, NO exerts protective and regenerative effects on the endothelial cells, inhibits proliferation and migration of the smooth muscle cells [29,30,31]; thus, it acts as an anti-atherosclerotic agent—if it can reach its physiological targets in the arterial wall to induce its physiological effects. Namely, when the redox balance in the vascular wall shifts towards the oxidative direction, reactive oxygen species (ROS), e.g., superoxide anion (O_2_^−^), transform NO into reactive nitrogen species (RNS) (in the case of O_2_^−^, into peroxynitrite) that results in nitrosative stress besides the oxidative one [32]. Although ROS are continuously generated even during the normal metabolism, ROS production, in response to all types of stress, shows a dramatic increase leading to the condition known as oxidative stress. Oxidative stress results in tissue damage and consequent inflammation, affecting a variety of metabolic pathways including NO homeostasis [33]. On one hand, expression of iNOS markedly increases followed by a substantial rise of NO (and O_2_^−^, see below) production. On the other hand, NOS isoforms (including eNOS) undergo a conformational change called uncoupling, as a result of which they will produce O_2_^−^ instead of NO [34]. Paradoxically, in spite of the increased NO production in oxidative stress, deficiency of NO is seen at the level of the physiological NO-induced pathways, because of the enhanced NO consumption during RNS formation. The above-mentioned processes highlight the crucial role of redox status in the evolution of CVD [35].

Reduced glutathione (GSH) is the most significant small antioxidant molecule in living organisms, accomplishing the elimination of ROS and RNS, reduction of lipid peroxides, and protection of SH– group containing enzymes from inactivation (via *S*-glutathionylation). During its reducing action, GSH turns into GSSG (oxidized glutathione (glutathione disulfide)), the oxidized form made through SH-bridge. GSH is transformed back in a NADPH-dependent reaction catalyzed by glutathione reductase, ensuring the redox balance in healthy organisms [36]. Deficiency of glutathione-related antioxidant defenses exists e.g., in atherosclerotic plaques [37].

Earlier studies have demonstrated that diosgenin influences the carbohydrate homeostasis at several molecular targets, being capable of effectively reducing hyperglycemia in pharmacologically induced rat diabetes models [38,39]. The underlying mechanisms are multifactorial and include restoration of pancreatic β-cell function, modulation of hepatic enzymes (regulating gluconeogenesis and antioxidant capacity of the liver) [40], promotion of adipocyte differentiation, inhibition of macrophage infiltration into adipose tissue, and decrease of expression of inflammatory genes [41]. It has been also observed that diosgenin decreases expression of the C/EBP homologous protein (CHOP) leading to reduced stress of endoplasmic reticulum in pancreatic islet β-cells. Moreover, diosgenin enhances expression of peroxisome proliferator-activated receptor gamma (PPRγ) that results in increased adipocyte differentiation, decreased adipocyte size and ectopic fat accumulation [38]. In addition, diosgenin, acting as a PPRγ agonist, may mimic the effect of thiazolidinediones, classic insulin-sensitizing drugs, in the peripheral tissues [42].

Disturbance to lipid synthesis and accumulation is a characteristic phenomenon in obesity and MetS that promotes development of atherosclerosis and CVD. Thus, maintenance or restoration of normal lipid homeostasis is a major goal in the therapy of MetS [43,44]. Lipid metabolism is controlled by sterol-regulatory, element-binding proteins (SREBPs), a family of membrane-bound transcription factors. SREBPs activate expression of several genes associated with lipid absorption, excretion and metabolism [45]. SREBP-1c is influenced by liver X receptor-a (LXRa), which functions primarily as a sterol sensor, with additional roles in bile acid synthesis and metabolism, cholesterol movement, fatty acid synthesis and lipoprotein metabolism [46]. It has been recently demonstrated that diosgenin can ameliorate dyslipidemia through inhibiting transactivation of LXRa in KK-Az mice [47]. This finding corroborates the observation that diosgenin increases levels of antioxidant and hepatoprotective enzymes in the blood plasma and liver, with concomitant improvement of β-cell regeneration and insulin secretion in streptozotocin-induced diabetic rats [40]. It has been also found that diosgenin increases the phosphorylation of AMP-activated protein kinase (AMPK) and acetyl-CoA carboxylase (ACC) in HepG2 cells, furthermore it suppresses LXRa in a rat model of non-alcoholic fatty liver disease (NAFLD), thus preventing development of NAFLD [48].

Ex vivo studies have revealed that diosgenin can directly modulate the function of arterial smooth muscle by regulating cell viability and blocking smooth muscle cell migration [49], furthermore it can evoke endothelium-dependent vasorelaxation that could be blocked by an eNOS inhibitor [50]. Moreover, in cultured human endothelial cells, diosgenin has been found to ameliorate FFA-induced endothelial dysfunction and insulin resistance via inhibiting the IKKβ and IRS-1 pathways [51].

Diosgenin is not the only phytochemical responsible for the beneficial effects of fenugreek. Fenugreek seed contains large amounts of amino acids, including 4-hydroxyisoleucine (4-OH-Ile) that stimulates insulin secretion and improves insulin sensitivity [52,53,54,55]. The underlying mechanism encompass enhancement of glucose uptake by translocation of GLUT4 (glucose transporter 4) to the plasma membrane [56]. Moreover, 4-OH-Ile also exerts anti-inflammatory action via reducing TNF-α mRNA expression [57,58]. Galactomannan, another component of fenugreek, inhibits some lipid- and carbohydrate-hydrolyzing enzymes, such as α-glucosidase, that leads to decreased lipid and glucose absorption [59,60,61]. These properties of fenugreek compounds have been commercialized in products for general use, such as Fenfuro^TM^, a patented dietary supplement containing fenugreek seed extract that has proven to be useful in the therapy of T2DM [62].

In association with the metabolic effects mentioned above, fenugreek seed and diosgenin both exhibit anti-inflammatory properties, the precise mechanism of which is not fully defined yet. Nevertheless, it has been observed that diosgenin inhibits expression of VCAM-1 (vascular cell adhesion molecule) and ICAM-1 (intracellular adhesion molecule), proteins involved in the pathogenesis of atherosclerosis, abolishes TNF-α induced production of intracellular ROS and inhibits NF-κB and IκB kinase activation, along with subsequent degradation of IκBα, and nuclear translocation of NF-κB. These findings show that diosgenin is a potent agent for inhibiting the formation and growth of atherosclerotic lesions [24]. Decrease of ROS formation by diosgenin is especially important, because almost all ROS-related pathologic processes involve transformation of NO into peroxynitrite that conversion diminishes the bioavailability of NO and leads to the condition called ED [32].

Our finding, i.e., ED can be an early sign of metabolic diseases, is in agreement with results of several previous studies. In an investigation using rabbits fed with high-fat diet (HFD) for 2–8 weeks, ED developed already at the second week [63]. High level of free fatty acids (FFAs) thought to play a prominent role to evoke ED, because FFAs downregulate the AMPK/PI3K/Akt/eNOS signaling pathway [64], activate the NADPH oxidase leading to enhanced ROS generation and oxidative stress [65], and also activate the protein kinase C and NF-κB pathways hampering the vascular insulin signaling [66]. ED has been found to possess a prognostic impact on recognizing CVD [67], moreover it can be considered as an independent risk factor of CVD [68,69]. Consistently, in our early stage MetS model (with six-week HFHSD), ED developed even at normal plasma glucose and insulin levels.

According to the classic concept of the regulation of arterial tension, one of the most important roles of the endothelium is to respond to chemical stimuli such as Ach, by release of vasodilatory agents, notably NO, which acts on adjacent smooth muscle cells to relax and increase diameter of the blood vessel [70,71]. However, a new vasodilatory concept has recently been proposed which is that arterial smooth muscle is more than just a passive recipient of endothelial NO, and in fact it is a paracrine effector that responds to Ach by release of NO, thereby contributing to vascular tone regulation [72]. In this scenario, the role of the endothelial layer is predominantly to protect the arterial media from oxidative stressors (such as superoxide anion) and thereby to maintain the appropriate redox balance in the tissue compartment of arterial smooth muscle [27,73]. This concept is supported by the observation that presence of effective superoxide scavengers (tempol and *N*-acetyl-l-cysteine) allowed the endothelium-deprived rat aorta sections to partially preserve their dilatory response capacity to Ach. This observation suggests that the major underlying mechanism, by which endothelial cells enable the so-called endothelium-dependent vasorelaxation, is to protect the smooth muscle-derived NO from oxidative stressors and to allow it to mediate a dilatory action [72].

**Figure A1:**
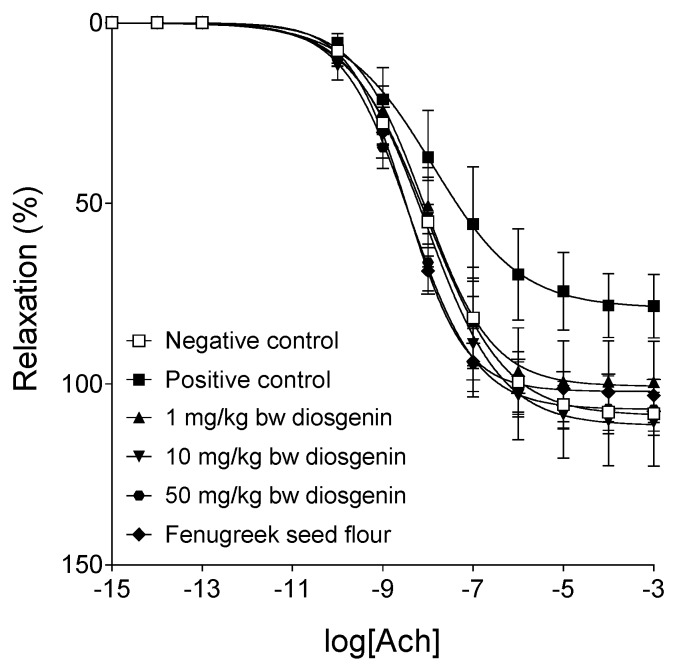
The relationship between the concentration of acetylcholine (Ach) and the NO-mediated (so-called endothelium-dependent) arterial relaxation in experimental groups subjected to different diets, created from real Ach concentration-response (E/c) curves presented on Figure 3. The axis *x* shows the common logarithm of molar concentration of Ach, and the axis *y* indicates the effect as a percentage decrease of the initial contractile force. The symbols show the modified effect values averaged within the groups (±SEM), and the lines denote the Hill equation fitted to the averaged modified Ach E/c data.

The precise mechanism of achieving a balance between vasoconstrictive and vasodilatory processes remains to be fully characterized. It nevertheless may be concluded that a complex relationship exists between endothelial function and redox conditions in the vasculature: the physiological endothelial function ensures the appropriate oxidoreductive state in the microenvironment of vascular smooth muscle (maintaining the intact smooth muscle responsiveness), while oxidative stress in the circulation damages the function of endothelial cells (shifting the redox balance in the media and thereby leading to weaker smooth muscle response to vasodilators). In this respect, results reported by the present study have provided further evidence in support of the significance of redox balance to sustain the normal dilatory susceptibility of arteries and thereby the physiological arterial tension. In addition, results of the present study demonstrate the significant antioxidant property of fenugreek seed compounds, especially that of diosgenin. Results of our functional assays indicate that dietary fenugreek seed flour as well as diosgenin (used in sufficiently high doses) can significantly improve, and indeed, practically restore the HFHSD-damaged arterial response to Ach, a classic vasodilatory signal molecule (Figure 3). Taking into consideration the functional findings reported here (Figure 3, Figure A1, Table A1) together with results of assays for blood redox status (Figure 4, Figure 5 and Figure 6), it is proposed that improvement of endothelium-dependent vasorelaxation in response to fenugreek and diosgenin treatment was mediated by the antioxidant action of diosgenin and possibly by other fenugreek components. The above findings and interpretations, nevertheless, raise an important question: why is it that decreased antioxidant capacity was not detected in the plasma of animals receiving only HFHSD (positive controls), along with elevated plasma antioxidant capacity in the fenugreek- and diosgenin-treated animals, in comparison with the negative controls (Figure 6)? A speculative account for these observations is that the redox balance perturbed by HFHSD may be more easily compensated in the bloodstream than in the arterial media. An alternative (but not exclusive) explanation may be that in response to HFHSD, a special kind of redox balance developed in the blood. This redox state, although it seemed to be normal according to outcomes of the assays reported here, was insufficient to maintain physiological endothelial function and/or the intact smooth muscle responsiveness. In turn, scavengers of reactive oxygen species (such as diosgenin) could substantially increase the antioxidant capacity in the arterial wall without significantly affecting the redox balance in the blood, which may be due to their lipophilic property.

In summary, both fenugreek and diosgenin can prevent the impairment of arterial endothelial function (characterized by the response to Ach) developed during HFHSD. This beneficial effect may be associated with the simultaneous decrease in plasma glutathione levels and glutathione reductase activity that may reflect the reduced need for endogenous antioxidants in the presence of exogenous ones. Although patterns of beneficial effects of fenugreek and diosgenin are quite similar, they show some moderate differences. Namely, at lower Ach levels, fenugreek can produce somewhat stronger enhancement in the response to Ach than diosgenin. In light of the present results, fenugreek seed preparations may play an important role in future supplement therapy of metabolic disorders (including MetS), even at their early stage with seemingly intact body weight and carbohydrate homeostasis.

## 4. Materials and Methods

### 4.1. Materials

The following chemicals and preparations were used: conventional rat food (S8106-S011 SM R/M-Z + H, obtained from Ssniff Spezialdiäten GmbH, Soest, Germany), high-fat rat food (rodent maintenance (RM) Atwater fuel energy (AFE) 45% fat, 20% crude protein (CP) and 35% carbohydrate (CHO), obtained from Special Diets Services, Witham, UK), fenugreek seed flour (obtained from Trigonella Med. Ltd., Mosonmagyarovar, Hungary), norepinephrine hydrochloride (NE; obtained from the institutional drugstore as vial Arterenol^®^, Sanofi-Aventis, Franfurt, Germany), furthermore diosgenin, acetylcholine chloride (Ach) and chemicals for Krebs solution (purchased from Sigma, St. Louis, MO, USA).

Krebs solution contained (in mmol/L): NaCl: 118, KCl: 4.7, CaCl2: 2.5, NaH2PO4: 1, MgCl2: 1.2, NaHCO3: 24.9, glucose: 11.5, and ascorbic acid: 0.1, dissolved in redistilled water. Krebs solution was used to dissolve Ach, and to dilute the NE solution.

### 4.2. Animals and Protocols

The investigation was approved by the Committee of Animal Research, University of Debrecen, Hungary (25/2013DEMÁB; date: 29 January 2014). Male Wistar rats weighting 300–500 g were randomly divided in six groups: negative control group receiving conventional rat food and tap water ad libitum (*n* = 6); positive control group receiving high-fat rat food and 5% *m*/*v* glucose solution ad libitum as high-fat, high-sugar diet (HFHSD) (*n* = 6); fenugreek group treated with 2 g/kg bw/day fenugreek seed flour (containing 10 mg/kg bw/day diosgenin) in addition to HFHSD (*n* = 9); diosgenin groups treated with 1, 10 and 50 mg/kg bw/day diosgenin in addition to HFHSD (*n* = 8, 8 and 8, respectively). Fenugreek and diosgenin were added to the chow (at the beginning of the HFHSD treatment). The daily dose was calculated on the basis of average daily food consumption of the animals.

After 6 weeks, the animals were guillotined. Plasma glucose level was determined from the blood using Accu-Chek Active meter (Roche Holding AG, Basel, Switzerland). Plasma insulin was measured using ELISA kit (DA-INSULIN-2015; Cortez Diagnostics Inc., Woodland Hills, CA, USA). In addition, blood was collected from each rat into EDTA-coated vacutainer tubes (BD, Franklin Lakes, NJ, USA) to determine plasma concentrations of glutathione, glutathione-handling enzymes and antioxidant capacity. The blood samples were centrifuged at 1000× *g* at 4 °C for 10 min. The plasma was divided into aliquots and stored at −20 °C until analysis.

### 4.3. Protocol of the Functional Assay

Immediately after guillotining (and collecting blood), the proximal part of the abdominal aorta was isolated, and 2 mm wide rings were cut off (two rings from each animal). The rings were mounted horizontally at 10 mN resting tension, using a wire instrument, in a 10-mL vertical organ chamber (Experimetria TSZ-04, Experimetria Ltd, Budapest, Hungary) containing Krebs solution, oxygenated with 95% O_2_ and 5% CO_2_ (36 °C; pH = 7.4). The isometric contractile force of the circulatory muscle layer was measured by a transducer (Experimetria SD-01) and strain gauge (Experimetria SG-01D), and recorded by a polygraph (Medicor R-61 6CH Recorder).

After a 60-min incubation period, a NE concentration-response (E/c) curve (from 0.1 nmol/L to 1 µmol/L) was generated on the aortic rings to determine their half maximal effective concentration (EC_50_) value. After 60-min wash-out, EC_50_ for NE was administered to the rings, and, after the stabilization of the contractile force at a higher value (pre-contraction), an Ach E/c curve was constructed (from 0.1 nmol/L to 1 mmol/L).

### 4.4. Determination of Reduced (GSH) and Oxidized (GSSG) Glutathione

GSH and GSSG were determined using a commercially available assay kit (kit number: 703002; Cayman Chemical, Ann Arbor, MI, USA). In the assay, 5,5′-dithio-bis (2-nitrobenzoic acid) (DTNB) and GSH react and generate a yellow product (2-nitro-5-thiobenzoic acid). Under the assay conditions, GSSG was reduced producing 2 molar equivalents of GSH. The absorbance was measured colorimetrically at 405 nm for both GSH and GSSG, and sample concentrations were determined by means of a standard GSH and GSSG curve expressing plasma concentrations in µmol/L (respectively).

### 4.5. Determination of Glutathione Peroxidase

Glutathione peroxidase activity was determined using a commercially available assay kit (kit number: ab102530; Abcam Plc., Cambridge, UK). In this assay, glutathione peroxidase generates GSSG from GSH during H_2_O_2_ reduction and the generated GSSG is reduced back to GSH by glutathione reductase during consumption of NADPH. The reduction of NADPH is proportional to glutathione peroxidase activity, thus, it can be measured colorimetrically at 340 nm. The kit reagents were dissolved as described in the protocol book. The standard curve was prepared as described in the kit. 90 µL of diluted standard solutions was added into the correct wells. 50 µL of samples, positive control and reagent control was added to the plate into the correct well. The reaction mixture was as described in the protocol book. The mix contained 33 µL of assay buffer, 3 µL of 40 mmol/L NADPH solution, 2 µL of glutathione reductase solution and 2 µL of GSH solution per well, except for standard wells. 40 µL of the reaction mix was added to the samples, positive control and reagent control. The plate was incubated at room temperature for 15 min. After incubation, absorbance was measured. If the absorbance value of the samples was lower than 1.0, then 1 µL of 40 mmol/L NADPH was added to the sample. 1 µL of 40 mmol/L NADPH gave an ~0.5 increase in absorbance at 340 nm. After this step, 10 µL of cumene hydroperoxide was added to the samples, positive control and reagent control wells to start the glutathione peroxidase reaction. The microplate reader was set to take 1 reading every 5 min for 30 min for glutathione reductase activity measurement. Measurements started immediately after the addition of cumene hydroperoxide at 340 nm:

Δ*A*_340nm_ = (sample *A*_1_ − sample *A*_2_) − (reagent control *A*_1_ − reagent control *A*_2_)

where *A*_1_ is the absorbance value in the first reading, *A*_2_ is the absorbance data in the second reading. An NADPH standard curve was plotted and Δ*A*_340nm_ was applied to this curve to obtain Δ*B* for NADPH. The obtained result was substituted into the equation:

$$\mathrm{glutathione}\;\mathrm{peroxidase}\;\mathrm{activity}=\frac{\Delta B}{\left(T_{2}-T_{1}\right)\;V}\;D$$

where Δ*B* is the amount of NADPH that decreased between *T*_1_ and *T*_2_ (in nmol), *T*_1_ is the time of the first reading (*A*_1_) (min), *T*_2_ is the time of second reading (*A*_2_) (min), *V* is the pre-treated sample volume added into the reaction well (mL), and *D* is the sample dilution factor. An NADPH standard curve was plotted, and equations suggested by the kit were used. The activity of glutathione peroxidase was expressed in nmol/min/mL = U/L.

### 4.6. Determination of Glutathione Reductase

Glutathione reductase activity was determined using a commercially available assay kit (kit number: ab83461; Abcam Plc., Cambridge, UK). In this assay, glutathione reductase forms GSH from GSSG, then, while GSH reacts with DTNB, a 2-nitro-5-thiobenzoate anion (TNB^2−^) is generated. By measuring the change in absorbance at 405 nm, glutathione reductase activity can be determined as follows:

$$\mathrm{glutathione}\;\mathrm{reductase}\;\mathrm{activity}=\frac{\Delta B}{\left(T_{2}-T_{1}\right)\;0.9V}\;D$$

where Δ*B* is the TNB amount from TNB standard curve (in nmol), *T*_1_ is the time of the first reading (*A*_1_) (in min), *T*_2_ is the time of the second reading (*A*_2_) (in min), *V* is the pre-treated sample volume added into the reaction well (in mL), 0.9 is the sample volume change factor during the sample pre-treatment procedure, and *D* is the dilution factor, as suggested in the kit’s description. Glutathione reductase activity was expressed in nmol/min/mL = U/L.

### 4.7. Determination of Antioxidant Capacity of Water-Soluble Compounds (ACW)

ACW was determined using a commercially available ACW kit (kit number: 846-60002-0; Analytik Jena AG, Jena, Germany). Radicals were generated photochemically by UV irradiation of a photosensitizer compound. PhotoChem measures the inhibition of radicals by the sample ACW content. The ACW kit contains R1, R2, R3 and R4 reagents. R1 and R2 are ready for use and stored at 2–8 °C. R3 is lyophilized and stored at −20 °C until use. 750 µL of R2 reagent was added into R3 and was ready to use. A stock solution was made from R4 reagent, 490 µL of R1 reagent and 10 µL of H_2_SO_4_ were added into R4 reagent. 10-fold dilutions were made from the stock solution (10 µL of R4 stock solution + 990 µL of R1 solution). First the blank was prepared: 1500 µL of R1, 1000 µL of R2, and 25 µL of R3 were mixed. A calibration was made between 0.1 and 2 nmol (at least 5 points) with l-ascorbic acid standard. The plasma sample was centrifuged at 10,000 rpm for 5 min and the supernatant of samples was used for measurements. The working solution contained 1490 µL of R1, 1000 µL of R2, 25 µL of R3 and 10 µL of samples. The calculation for the lyophilized sample is:

$$\mathrm{concentration}=\frac{\left(\mathrm{results})\;(\mathrm{dilution})\;(176.13\right)}{\mathrm{pipetted}\;\mathrm{volume}}$$

The “result” is the result by the PhotoChem, 176.13 is the molecular weight of l-ascorbic acid, pipetted volume is the sample volume (in µL). The final result was expressed as µg/mL l-ascorbic acid equivalent.

### 4.8. Determination of Antioxidant Capacity of Lipid-Soluble Compounds (ACL)

ACL was determined using a commercially available ACL kit (kit number: 849-60004-0; Analytikjena AG). The method is similar to that used for ACW test. The ACL kit contain R1, R2, R3, and R4 reagents. R1 and R2 were ready for use but stored at 2–8 °C. R3 was lyophilized and stored at −20 °C until use. 750 µL of R2 reagent was added into R3 and was ready to use. Stock solution was made from R4 reagent: 500 µL of R1 reagent was added into R4 reagent, and 10-fold dilutions were prepared from the stock solution (10 µL of R4 stock solution +990 µL of R1 solution. First the blank was prepared: 2300 µL of R1, 200 µL of R2, and 25 µL of R3 were mixed. A calibration was made between 0.2 nmol and 3 nmol (at least 5 points) with a trolox standard. The sample was centrifuged at 10,000× *g* for 5 min. The supernatant of prepared samples was use for measurements. The working solution contained 2290 µL of R1, 200 µL of R2, 25 µL of R3 and 10 µL of the samples. The calculation for the lyophilized sample is:

$$\mathrm{concentration}=\frac{\left(\mathrm{results})\;(\mathrm{dilution})\;(250.3\right)}{\mathrm{pipetted}\;\mathrm{volume}}$$

The “result” means the result by the PhotoChem, 250.3 is the molecular weight of trolox, pipetted volume is the sample volume (in µL). The final result was expressed as µg/mL trolox equivalent.

### 4.9. Data Processing and Analysis

Responses of aortic rings obtained from the same rat were averaged.

The effect of NE was defined as an (absolute) increase in addition to the resting tension (10 mN) of aortic rings.

If relaxation occurred in the presence of a given Ach concentration, the maximal relaxation was considered. If the ring produced a contraction, its maximum was taken into account. The effect of Ach was defined as a percentage change in the initial tension of aortic rings (after the pre-contraction with NE using their EC_50_ value).

Normality of data sets was verified with D’Agostino–Pearson omnibus test. Two data sets were compared with unpaired, two-tailed Student’s *t* test or Welch’s *t* test (if variances were not equal). More than two data sets were compared using one-way ANOVA (with Geisser–Greenhouse correction) followed by Tukey post-testing. Difference of means was considered significant at *p* < 0.05. Statistical analysis was carried out with GraphPad Prism 7.04 (GraphPad Software Inc., La Jolla, CA, USA).

## Abbreviations

4-OH-Ile	4-hydroxyisoleucine
ACC	acetyl-CoA carboxylase
Ach	acetylcholine
ACL	lipid soluble antioxidant capacity
Act	protein kinase B (PKB)
AMPK	adenosine monophosphate-activated protein kinase
AWS	water soluble antioxidant capacity
bw	body weight
cGMP	cyclic guanosine monophosphate
CHOP	CCAAT-enhancer-binding homologous protein
CVD	cardiovascular diseases
ED	endothelial dysfunction
eNOS	endothelial nitric oxide synthase
FFA	free fatty acid
GLUT4	glucose transporter 4
GSH	reduced glutathione (glutathione)
GSSG	oxidized glutathione (glutathione disulfide)
HDL	high-density lipoprotein
HFHSD	high-fat, high-sugar diet
ICAM	intracellular adhesion molecule
IKK-β	inhibitor of nuclear factor kappa-B kinase subunit beta
iNOS	inducible nitric oxide synthase
IRS1	insulin receptor substrate 1
IκB	inhibitor of kappa B
LDL	low-density lipoprotein
LXRa	liver X receptor-a
MAPK	mitogen-activated protein kinase
MetS	metabolic syndrome
mRNA	messenger ribonucleic acid
NAFLD	non-alcoholic fatty liver disease
NE	norepinephrine
NF-κB	nuclear factor kappa-light-chain-enhancer of activated B cells
nNOS	neuronal nitric oxide synthase
NO	nitric oxide
NOS	nitric oxide synthase
PGI2	prostacyclin
PI3K	phosphatidylinositol-4,5-bisphosphate 3-kinase
PPRγ	peroxisome proliferator-activated receptor gamma
RAAS	renin-angiotensin-aldosterone system
RNS	reactive nitrogen species
ROS	reactive oxygen species
SEM	standard error of the mean
SREBPs	sterol regulatory element-binding proteins
T2DM	type 2 diabetes mellitus
TNF-α	tumor necrosis factor alpha
VCAM	vascular cell adhesion molecule

## Appendix A

For the evaluation of an E/c curve, nonlinear regression (curve fitting) is a common method [74]. However, Ach E/c curves of the present investigation, in their original form, were not suitable for curve fitting, because two opposite responses were evoked by Ach: a relaxation mediated by NO (synthesized in the endothelial and perhaps arterial smooth muscle cells), and an NO-independent contraction. Although, due to its greater EC_50_ value, this latter effect developed with a considerable delay along the axis *x*, there was a significant overlap between the two opposite responses (as a resultant, see Figure 3) that hindered the reliable fitting of the Hill equation, the most common choice to assess an E/c relationship [75].

In order to quantify separately the NO-dependent relaxation and to make the regression more reliable, Ach E/c data cleansing and manipulation were performed as follows. For each individual Ach E/c curve (resulted from averaging of data of the two aortic rings obtained from the same animal), the maximal effect value was chosen and arbitrarily assigned to all Ach concentrations that were higher than the Ach concentration, to which the maximal effect belonged. Furthermore, zero effect was assigned to three surely effectless Ach concentration values (1, 10, 100 fmol/L), and these E/c data were arbitrarily added to each individual Ach E/c data set. These modified Ach E/c curves were fitted to the Hill equation (in the GraphPad Prism software, the four-parameter log[agonist] vs. response equation with a bottom constrained to zero).

The fenugreek- and diosgenin-treated aortic rings produced practically the same Ach-induced arterial relaxation as the negative control aortic rings (representing the healthy state), and they all showed more pronounced dilatory response to Ach than the positive control preparations (reflecting the ED in an early-stage MetS) (Figure A1).

The E_max_, logEC_50_ and n (Hill coefficient) parameters provided by the Hill equation (fitted to the individual modified Ach E/c data) were compared among the different experimental groups. Only differences of E_max_ values reached the level of statistical significance: E_max_ of the negative control group, 50 mg diosgenin group and fenugreek group significantly exceeded that of the positive control group (receiving HFHSD) (Table A1), in agreement with the results of analysis of real Ach E/c curves (Figure 3). Additionally, ED in the positive control group reflected in a moderately increased EC_50_, while beneficial effect of diosgenin and fenugreek prevented the deleterious action of HFHSD. Moreover, treatments with the highest diosgenin dose and fenugreek decreased EC_50_ even as compared to the negative control. The Hill coefficient values of the groups varied between 0.64 and 0.73, excepting the fenugreek group, in which it increased to 0.85 (maybe as a results of the complex effect of different fenugreek components on the signaling pathways of Ach in the arterial wall) (Table A1). Our results yielded by curve fitting corroborate the findings obtained from the comparison of effect values of the intact Ach E/c curves (Figure 3).

**Table A1:** Regression parameters (expressed in mean ± SEM) of the Hill equation fitted to the individual modified acetylcholine (Ach) concentration-response (E/c) curves. Level of statistical significance for differences between the positive vs. negative control groups (asterisk), and between the diosgenin and fenugreek treated groups vs. positive control group (hash mark) is indicated (*, #: *p* < 0.05).

	Co−	Co+	1D	10D	50D	FSF
E_max_	108.11 ± 6.05	79.66 ± 8.6 *	100.58 ± 11.31	111.12 ± 11.9	107.80 ± 5.56 #	101.84 ± 4.43 #
logEC_50_	−7.94 ± 0.32	−7.65 ± 0.54	−7.95 ± 0.25	−7.95 ± 0.23	−8.3 ± 0.23	−8.45 ± 0.14
*n*	0.67 ± 0.07	0.68 ± 0.07	0.73 ± 0.08	0.65 ± 0.07	0.64 ± 0.04	0.85 ± 0.1

Co−: negative control group (receiving commercial rat diet); Co+: positive control group (receiving high-fat, high-sugar diet (HFHSD)); 1D, 10D and 50D: groups treated with 1, 10 and 50 mg/kg bw/day diosgenin in addition to HFHSD, respectively; FSF: group treated with 2 g/kg bw/day fenugreek seed flour (containing 10 mg/kg bw/day diosgenin); E_max_: maximal effect; logEC_50_: common logarithm of EC_50_, the concentration producing half-maximal effect; *n*: Hill coefficient (slope factor).
